# Supplementary material for: Exploring the Onset and Progression of Prostate Cancer through a Multicellular Agent-based Model
Source: Cancer Res Commun. 2023 Aug 7;3(8):1473–85. doi: 10.1158/2767-9764.CRC-23-0097 (PMC10405859; doi:10.1158/2767-9764.CRC-23-0097)
Supplement: Supplementary Figure 1 — Experimental data and model parameter fitting [file crc-23-0097-s01.pdf]

Supplementary Figure 1. *Experimental data and model parameter fitting*

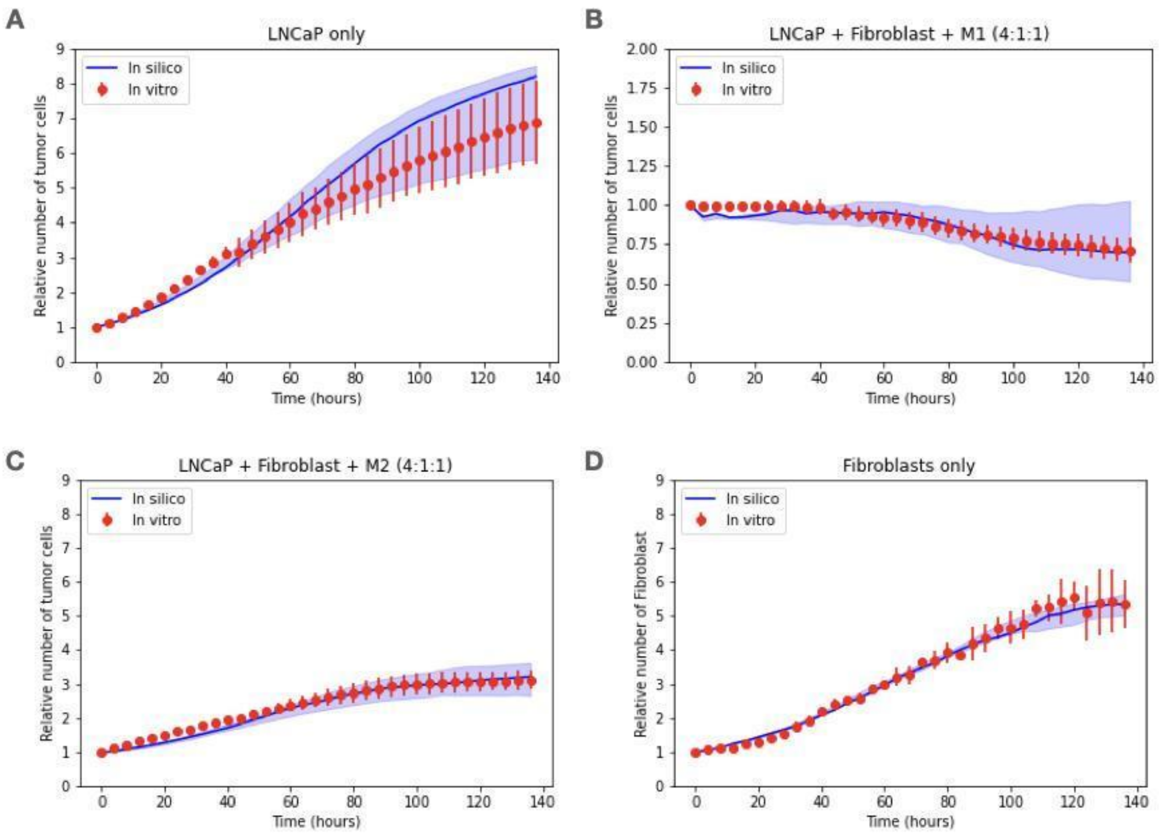

Supplementary Figure 1. Experimental data and model fitting. Growth curves of ***A****. LNCaP cells;* ***B****. LNCaP co-cultured with fibroblasts and M1 macrophages;* ***C****. LNCaP co-cultured with fibroblasts and M1 macrophages;* ***D****. Fibroblasts. Red dots represent means and error bars represent standard deviation of the three biological replicates (spanning six technical replicates). Blue lines represent model simulations using the median of the parameters estimated for the biological replicates, and shading represents the interquartile range for the 50 optimisations per biological replicates.*
