# Supplementary material for: Exploring the Onset and Progression of Prostate Cancer through a Multicellular Agent-based Model
Source: Cancer Res Commun. 2023 Aug 7;3(8):1473–85. doi: 10.1158/2767-9764.CRC-23-0097 (PMC10405859; doi:10.1158/2767-9764.CRC-23-0097)
Supplement: Supplementary Figure 2 — Comparison of cancer and non-cancer simulations [file crc-23-0097-s02.pdf]

***Supplementary Figure 2.*** *Comparison of cancer and non-cancer simulations*

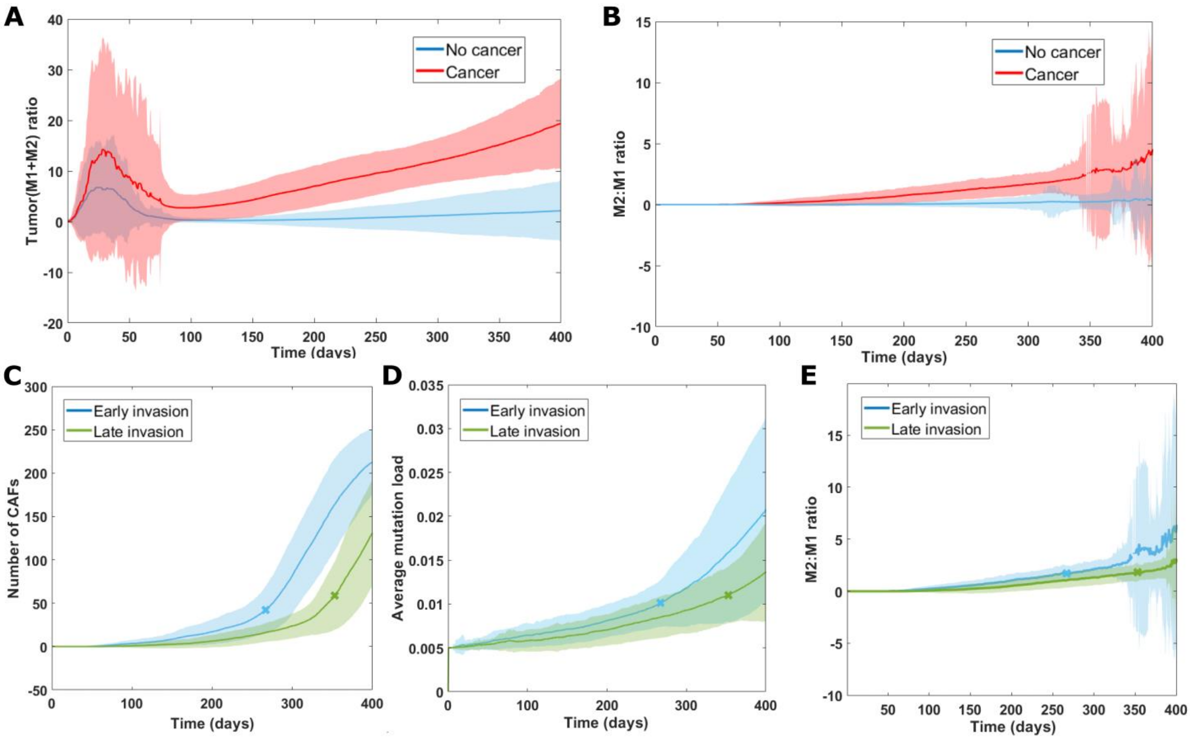


*Supplementary Figure 2. Cancer and non cancer simulation comparison. Average results (including standard deviation in the shaded area) for simulations using the standard parameter values (as described in table S2).* ***A****. Tumor cell to macrophage ratio (M1+M2) both for the simulations that resulted in cancer (180/500) and that did not result in cancer (320/500).* ***B****. Ratio of M2:M1 macrophages for non-cancer and cancer cases.* ***C****. Number of CAFs over time for all cancer cases in groups of early and late invasion (invasion time indicated with a cross).* ***D****. Average mutational load for all cancer cases of early and late invasion (invasion time indicated with a cross).* ***E****. M2:M1 ratio for all cancer cases (invasion time indicated with a cross).*
