# Supplementary material for: Exploring the Onset and Progression of Prostate Cancer through a Multicellular Agent-based Model
Source: Cancer Res Commun. 2023 Aug 7;3(8):1473–85. doi: 10.1158/2767-9764.CRC-23-0097 (PMC10405859; doi:10.1158/2767-9764.CRC-23-0097)
Supplement: Supplementary Figure 3 — Sensitivity analysis of the most sensitive model parameters [file crc-23-0097-s03.pdf]

Supplementary Figure 3. *Sensitivity analysis most sensitive model parameters*

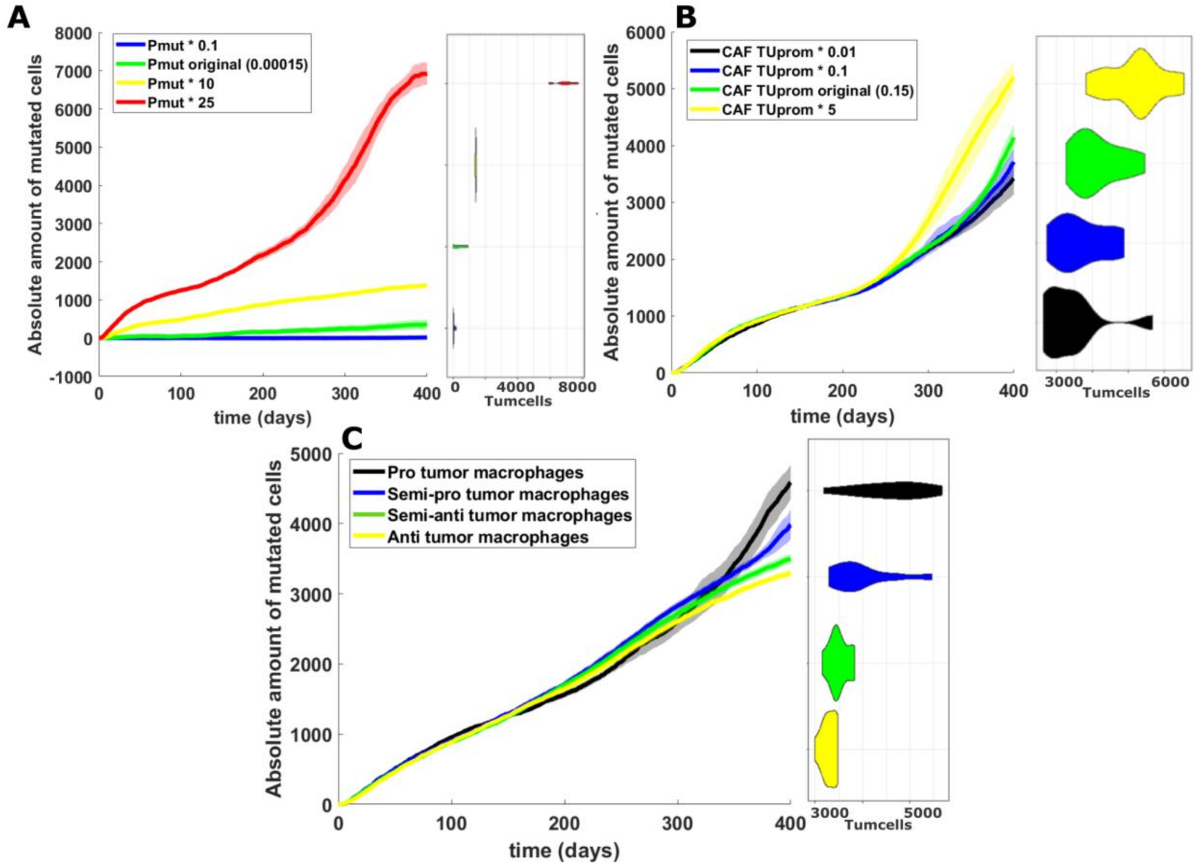

Supplementary Figure 3. Follow-up sensitivity analysis. ***A****. Sensitivity analysis of the probability to gain mutations for luminal cells. Next to this graph, the violin plot on the four different simulations is displayed.* ***B****. Sensitivity analysis of the probability for mutated cell proliferation promotion by CAFs. The violin plot is displayed next to the graph, the colors correspond to those displayed in the graph.* ***C.*** *Sensitivity analysis of the combined parameters for macrophages (M1 migration probability and mutation threshold). Pro-tumor macrophages have a lower threshold for transition towards pro-tumor phenotype and larger migration probability. Next to the graph, the violin plot for the four simulations is displayed*
