# Supplementary material for: Exploring the Onset and Progression of Prostate Cancer through a Multicellular Agent-based Model
Source: Cancer Res Commun. 2023 Aug 7;3(8):1473–85. doi: 10.1158/2767-9764.CRC-23-0097 (PMC10405859; doi:10.1158/2767-9764.CRC-23-0097)
Supplement: Supplementary Figure 4 — Pairwise combinations of the most sensitive model parameters [file crc-23-0097-s04.pdf]

Supplementary Figure 4. *Pairwise combinations of most sensitive model parameters*

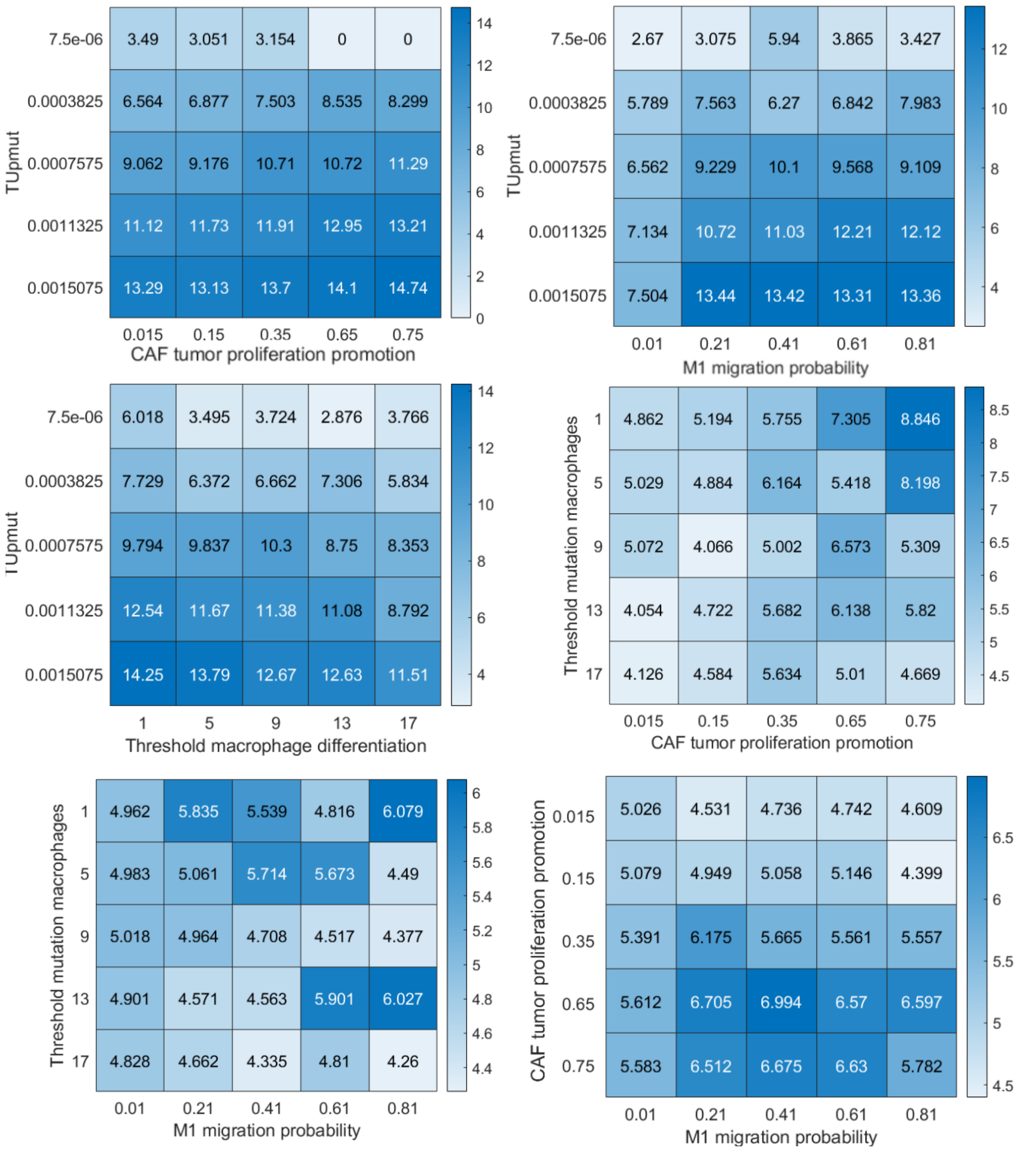

Supplementary Figure 4. Pairwise combinations. Heatmaps of the pairwise combinations of selected model parameters. The darker the square (and higher the number), the larger the tumor. The numbers indicate relative tumor sizes.
