# Supplementary material for: Exploring the Onset and Progression of Prostate Cancer through a Multicellular Agent-based Model
Source: Cancer Res Commun. 2023 Aug 7;3(8):1473–85. doi: 10.1158/2767-9764.CRC-23-0097 (PMC10405859; doi:10.1158/2767-9764.CRC-23-0097)
Supplement: Supplementary Table 1 — List of model assumptions [file crc-23-0097-s05.pdf]

Supplementary Table 1. *List of model assumptions*

Model assumption	Reference
The shape and ratios of cells in the starting geometry; a prostatic acinus	(1)
The prostatic acinus is surrounded by ECM	(2)
ECM has variable permeability, depending on the stage of the disease	(2)
The presence and percentage of prostate cancer stem cells	(3,4)
Tissue resident fibroblasts are quiescent and become active upon receiving a signal	(5)
Basement membrane is quiescent and functions as a barrier between the inside and outside of the acinus.	(1,6)
Quiescent cells remain in equilibrium/homeostasis; they do not migrate, proliferate or die	Trivial
Basement membrane is broken down by proteolytic enzymes produced by tumor cells. Not all enzyme production results in actual break down (only 10-30 percent)	(6)
Basement membrane break down does not happen right away, so tumor cells have to gain a certain amount of mutations before they can start break down	(6)
Basal cells and non-mutated luminal cells are in homeostasis; they can die and proliferate at low rates, within their normal/healthy regions	Trivial
Basal cells should always be attached to the basement membrane	(6)
Basal cells can only die after enough luminal cells have mutated	(7)
Non-quiescent cells can spontaneously enter apoptosis and die	(8), Experiments
Luminal cells can gain mutations	(1)
Mutated luminal cells have a higher chance of gaining another mutation and are not quiescent or in equilibrium	(1)
Luminal stem cells can also gain mutations, generating ’cancer stem cells’	(1)
Mutated cells can proliferate	Experiments
Upon gaining a mutation, oncogenic or tumor suppressing genes are affected. Mutated cells gain a proliferative advantage	(9)
Apart from the ’standard’ actions proliferate,migrate and die, mutated cells can perform additional actions (parallel to the other actions) once they have gained enough mutations	Trivial
Additional actions of mutated cells include: affecting differentiation of macrophages, breaking down basement membrane and affecting ECM permeability	(2,6,10)

Mutated cells can only break down basement membrane if they are directly adjacent to it	Assumption
Mutated cells move by a random walk and migrate towards fibroblasts	Experiments, (11)
Activation of tissue resident fibroblasts happens during PIN and is affected by mutated cells. CAFs co-evolve during disease progression.	(12–15)
The effect of CAFs on tumor cells depends on the distance between the two	(16)
CAFs can migrate towards mutated cells	(8)
CAFs can proliferate and migrate more than normal fibroblasts	(5)
CAFs stimulate primary tumor growth	(17)
CAFs elicit Epithelial Mesenchymal Transition (EMT) in mutated cells	(18)
CAFs modify the ECM; making it rather impermeable at the beginning and produce MMPs later on to degrade it	(2,13,19)
Macrophages cannot proliferate (the few times they possibly could does not affect simulations)	Experiments
M1 macrophages enter the simulation from one point, mimicking entry via a blood vessel	(20)
Macrophages migrate towards mutated cells, but only if they are in reach (17 grid spaces). If they are further away, they move randomly	(8,19)
Macrophages infiltrate prostatic acini during PIN	(21,22)
Macrophages can kill mutated cells. M1 polarized macrophages are more likely to kill than M2 polarized macrophages	Experiments, (23)
If a macrophage kills a mutated cell, more macrophages are drawn to the acinus (increased influx)	(21,22)
M2 macrophages do not enter the simulation via the blood, but have to arise from differentiation of the M1 macrophages	(20)
M2 polarized macrophages promote mutated cell proliferation	Experiments, (20,21)
Macrophages have to be adjacent to mutated cells to be able to promote those or kill those	(21,24)
M2 macrophages can elicit anchorage independent growth in tumor cells	(21,25)

Supplementary Table 1. Assumptions that were made during model development, including their source.

References

Packer JR, Maitland NJ. The molecular and cellular origin of human prostate cancer. Biochim Biophys Acta. 2016;1863:1238–60.

2. Winkler J, Abisoye-Ogunniyan A, Metcalf KJ, Werb Z. Concepts of extracellular matrix remodelling in tumour progression and metastasis. Nat Commun. 2020;11:5120.
3. Collins AT, Berry PA, Hyde C, Stower MJ, Maitland NJ. Prospective identification of tumorigenic prostate cancer stem cells. Cancer Res. 2005;65:10946–51.

4. Zhang K, Guo Y, Wang X, Zhao H, Ji Z, Cheng C, et al. WNT/β-Catenin Directs Self-Renewal Symmetric Cell Division of hTERThigh Prostate Cancer Stem Cells [Internet]. Cancer Research. 2017. page 2534–47. Available from: http://dx.doi.org/10.1158/0008-5472.can-16-1887
5. Kalluri R. The biology and function of fibroblasts in cancer. Nat Rev Cancer. 2016;16:582–98.
6. Liu A, Wei L, Gardner WA, Deng C-X, Man Y-G. Correlated alterations in prostate basal cell layer and basement membrane. Int J Biol Sci. 2009;5:276–85.
7. Bostwick DG, Brawer MK. Prostatic intra-epithelial neoplasia and early invasion in prostate cancer. Cancer. 1987;59:788–94.
8. Kather JN, Poleszczuk J, Suarez-Carmona M, Krisam J, Charoentong P, Valous NA, et al. Modeling of Immunotherapy and Stroma-Targeting Therapies in Human Colorectal Cancer. Cancer Res. 2017;77:6442–52.
9. Barbieri CE, Baca SC, Lawrence MS, Demichelis F, Blattner M, Theurillat J-P, et al. Exome sequencing identifies recurrent SPOP, FOXA1 and MED12 mutations in prostate cancer. Nat Genet. 2012;44:685–9.
10. Cess CG, Finley SD. Multi-scale modeling of macrophage-T cell interactions within the tumor microenvironment. PLoS Comput Biol. 2020;16:e1008519.
11. Erdogan B, Ao M, White LM, Means AL, Brewer BM, Yang L, et al. Cancer-associated fibroblasts promote directional cancer cell migration by aligning fibronectin. J Cell Biol. 2017;216:3799–816.
12. Bonollo F, Thalmann GN, Kruithof-de Julio M, Karkampouna S. The Role of Cancer-Associated Fibroblasts in Prostate Cancer Tumorigenesis. Cancers [Internet]. 2020;12. Available from: http://dx.doi.org/10.3390/cancers12071887
13. Ojalill M, Virtanen N, Rappu P, Siljamäki E, Taimen P, Heino J. Interaction between prostate cancer cells and prostate fibroblasts promotes accumulation and proteolytic processing of basement membrane proteins. Prostate. 2020;80:715–26.
14. Farhood B, Najafi M, Mortezaee K. Cancer-associated fibroblasts: Secretions, interactions, and therapy. J Cell Biochem. 2019;120:2791–800.
15. Tuxhorn JA, Ayala GE, Smith MJ, Smith VC, Dang TD, Rowley DR. Reactive stroma in human prostate cancer: induction of myofibroblast phenotype and extracellular matrix remodeling. Clin Cancer Res. 2002;8:2912–23.
16. Ghajar CM, Correia AL, Bissell MJ. The Role of the Microenvironment in Tumor Initiation, Progression, and Metastasis [Internet]. The Molecular Basis of Cancer. 2015. page 239–56.e5. Available from: http://dx.doi.org/10.1016/b978-1-4557-4066-6.00016-0
17. Linxweiler J, Hajili T, Körbel C, Berchem C, Zeuschner P, Müller A, et al. Cancer-associated fibroblasts stimulate primary tumor growth and metastatic spread in an orthotopic prostate cancer xenograft model. Sci Rep. 2020;10:12575.
18. Giannoni E, Bianchini F, Masieri L, Serni S, Torre E, Calorini L, et al. Reciprocal Activation of Prostate Cancer Cells and Cancer-Associated Fibroblasts Stimulates Epithelial-Mesenchymal Transition and Cancer Stemness [Internet]. Cancer Research. 2010. page 6945–56. Available from: http://dx.doi.org/10.1158/0008-5472.can-10-0785
19. Norton K-A, Jin K, Popel AS. Modeling triple-negative breast cancer heterogeneity: Effects of stromal macrophages, fibroblasts and tumor vasculature. J Theor Biol. 2018;452:56–68.
20. Jenkins SJ, Ruckerl D, Cook PC, Jones LH, Finkelman FD, van Rooijen N, et al. Local macrophage proliferation, rather than recruitment from the blood, is a signature of TH2 inflammation. Science. 2011;332:1284–8.
21. Thomas MU, Messex JK, Dang T, Abdulkadir SA, Jorcyk CL, Liou G-Y. Macrophages expedite cell proliferation of prostate intraepithelial neoplasia through their downstream target ERK. FEBS J. 2021;288:1871–86.
22. Fang L-Y, Izumi K, Lai K-P, Liang L, Li L, Miyamoto H, et al. Infiltrating macrophages promote prostate tumorigenesis via modulating androgen receptor-mediated CCL4-STAT3 signaling. Cancer Res. 2013;73:5633–46.
23. Sica A, Larghi P, Mancino A, Rubino L, Porta C, Totaro MG, et al. Macrophage polarization in tumour progression. Semin Cancer Biol. 2008;18:349–55.
24. Skvortsov S, Skvortsova I-I, Tang DG, Dubrovska A. Concise Review: Prostate Cancer Stem Cells: Current Understanding. Stem Cells. 2018;36:1457–74.
25. Cioni B, Zaalberg A, van Beijnum JR, Melis MHM, van Burgsteden J, Muraro MJ, et al. Androgen receptor signalling in macrophages promotes TREM-1-mediated prostate cancer cell line migration and invasion. Nat Commun. 2020;11:4498.
