# Supplementary material for: Exploring the Onset and Progression of Prostate Cancer through a Multicellular Agent-based Model
Source: Cancer Res Commun. 2023 Aug 7;3(8):1473–85. doi: 10.1158/2767-9764.CRC-23-0097 (PMC10405859; doi:10.1158/2767-9764.CRC-23-0097)
Supplement: Supplementary Table 2 — List of model parameters [file crc-23-0097-s06.pdf]

Supplementary Table 2. *List of model parameters*

Model parameter	Description	Value	Reference
LCpprol	Proliferation probability for luminal cells	0.3054	Experiments, A
TUpprol	Proliferation probability for mutated luminal cells	0.3054	Experiments, A
TUpmig	Migration probability for luminal cells that went through EMT	0.3501	Experiments, (1)
LCpdeath	Probability of death for luminal cells	0.00284	Experiments, A
TUpdeath	Probability of death for mutated luminal cells	0.00284	Experiments, A
TUrwalk	Random walk effect on movement	0.5	Experiments, (1)
TUpmax	Maximum amount of times luminal cells can proliferate (without mutations)	4	Experiments, A
TUps	Probability of symmetric division if proliferation for stem cells	0.2967	B
TUpmut	Probability of gaining a mutation for luminal cells	0.00015	(2), B
TUantig	Added antigenicity per mutation	0.005	B
TUmutmax	Maximum amount of antigenicity luminal cells can obtain	1	B
TUadded values	Added probability for gaining a mutation (for cells that have one or more mutations already)	0.15*TUantig	(2)
TUadded values	Added maximum proliferation capacity (eac time an additional mutation is gained)	0.75	B
TUadded values	Added proliferation probability	0.001* TUantig	B
TUpkill	Probability of breaking down the basement membrane by mutated cells	0.3	(3), B
TUthrshBM	Needed amount of mutations before basement membrane can be broken down	6	Arbitrary, C
TUpmdiff	Probability of affecting macrophage differentiation by mutated luminal cells	0.8	(4,5)
TUthrshM	Needed amount of mutations before macrophage differentiation can be affected by mutated cells	14	Arbitrary, C
TUpMMP	Probability of breaking down surrounding ECM	0	Variable, D

TUthrshMMP	Needed amount of mutations before mutated cells can affect ECM break down	15	Arbitrary, C
M1kmax	Maximum killing capacity of M1 macrophages	11	Experiments, A
M1pkill	Probability of killing by M1 macrophages	0.0918	Experiments, A
M1pmig	Migration probability of M1 macrophages	0.8001	(2)
M1pdeath	Probability of death for M1 macrophages	0.0147	Experiments, A
M1rwalk	Random walk effect on movement of M1 macrophages (if further away than 17 gridspaces)	0.8	(6), B
M1speed	Speed of movement	40	(2), B
M1engagement Duration	Number of steps the M1 cell is engaged	60	(2), B
M1influxRate	Amount of macrophages that enter the simulation each time	1	(1)
M1influxProb	Probability of M1 macrophages entering the simulation	0.08	B
M1influxadd	Factor with which the influx increases after killing the first mutated cell	3	B
M2kmax	Maximum killing capacity of M2 macrophages	11	Experiments, A
M2pkill	Probability of killing by M2 macrophages	0.0381	Experiments, A
M2pmig	Migration probability of M2 macrophages	0.8001	(2)
M2pdeath	Probability of death for M2 macrophages	0.0147	Experiments, A
M2rwalk	Random walk effect on movement of M2 macrophages (if further away than 17 gridspaces)	0.8	(6), B
M2speed	Speed of movement	40	(2), B
M2influxProb	Probability of M2 macrophages entering the simulation	0	(5)
M2TUadd	Added probability for proliferation of luminal cells	0.2985	Experiments, A
M2engagement duration	Number of steps the M2 cell is engaged	60	(2), B
M2emt	Probability of the M2 macrophage eliciting EMT in a mutated luminal cell if in range	0.75	(7), B
Fpprol	Proliferation probability of fibroblasts	0	(11), E
Fpmig	Migration probability of fibroblasts	0	(11), E
Fpdeath	Probability of death of fibroblasts	0	(11), E

Fpmax	Maximum proliferation capacity	0	(11), E
Frwalk	Random walk effect on movement of fibroblasts	0	(11), E
Fdiff	Probability of fibroblasts turning into CAFs when in range of mutated luminal cells	0.75	(8)
CFpprol	Proliferation probability of activated fibroblasts (CAFs)	0.00838	Experiments, B
CFpmig	Migration probability of CAFs	0.2	B
CFpdeath	Probability of death of CAFs	0.0054	(11), Experiments
CFpmax	Maximum proliferation capacity	4	Experiments, A
CFrwalk	Random walk effect on movement of CAFs	0.5	Experiments, B
CFemt	Probability of the CAF eliciting EMT in a mutated luminal cell if in range	0.75	(9),B
CFprom	Added probability for proliferation of mutated luminal cells	0.5* TUpprol	(10)
CFmmp	Probability of surrounding ECM break down by CAFs	0.9	B
CFmdiff	Probability of CAFs affecting macrophage differentiation	0.5	Variable, D
Cpprol	Proliferation probability of basal cells	0.2	(12), F
Cpmig	Migration probability of basal cells	0	(12), F
Cpdeath	Probability of death of basal cells	0.01	(12), F
Cpmax	Maximum proliferation capacity	10	B
Bpdeath	Probability of death of basement membrane	0	Trivial
Bpprol	Probability of proliferation for basement membrane	0	Trivial

*Supplementary Table 2. Model parameter values **A** -These parameters were determined by fitting them to the obtained experimental data, using the PSO algorithm described in the Materials and Methods section. **B** -These parameters can be freely chosen by the user. They were qualitatively tuned for these simulations to yield realistic results. **C** -These parameters (thresholds) can be freely chosen by the user. Increasing these parameters increases the amount of time it takes before disease can progress towards the next stage. **D** -These parameters were set to zero for the performed simulations, but can be set to higher values if the user wishes to include these processes/effects. **E** -These fibroblast parameters were all set to zero, as the tissue resident fibroblasts are believed to be quiescent before they are activated as CAFs in this case (11). **F** -Disruption of the basal cell layer takes place exponentially; only 0.7 percent breakdown in the first stage of PIN, 15 percent in the second stage and 52 percent in the third stage. These parameters were qualitatively tuned to get values close to these. **G** -Distances from which mutated cells affect macrophage differentiation and elicit ECM break down, the distances from which CAFs can break down ECM or elicit EMT and the distance from which macrophages can elicit*

anchorage independent growth were found to be insensitive in the sensitivity analysis. The chosen values are trivial (if bigger than 1).

References

1. Kather JN, Poleszczuk J, Suarez-Carmona M, Krisam J, Charoentong P, Valous NA, et al. Modeling of Immunotherapy and Stroma-Targeting Therapies in Human Colorectal Cancer. Cancer Res. 2017;77:6442–52.
2. Kather JN, Charoentong P, Suarez-Carmona M, Herpel E, Klupp F, Ulrich A, et al. High-Throughput Screening of Combinatorial Immunotherapies with Patient-Specific Models of Metastatic Colorectal Cancer. Cancer Res. 2018;78:5155–63.
3. Liu A, Wei L, Gardner WA, Deng C-X, Man Y-G. Correlated alterations in prostate basal cell layer and basement membrane. Int J Biol Sci. 2009;5:276–85.
4. Cess CG, Finley SD. Multi-scale modeling of macrophage-T cell interactions within the tumor microenvironment. PLoS Comput Biol. 2020;16:e1008519.
5. Wells DK, Chuang Y, Knapp LM, Brockmann D, Kath WL, Leonard JN. Spatial and functional heterogeneities shape collective behavior of tumor-immune networks. PLoS Comput Biol. 2015;11:e1004181.
6. Thomas MU, Messex JK, Dang T, Abdulkadir SA, Jorcyk CL, Liou G-Y. Macrophages expedite cell proliferation of prostate intraepithelial neoplasia through their downstream target ERK. FEBS J. 2021;288:1871–86.
7. Cioni B, Zaalberg A, van Beijnum JR, Melis MHM, van Burgsteden J, Muraro MJ, et al. Androgen receptor signalling in macrophages promotes TREM-1-mediated prostate cancer cell line migration and invasion. Nat Commun. 2020;11:4498.
8. Anderberg C, Pietras K. On the origin of cancer-associated fibroblasts. Cell Cycle. 2009;8:1461–2.
9. Giannoni E, Bianchini F, Masieri L, Serni S, Torre E, Calorini L, et al. Reciprocal Activation of Prostate Cancer Cells and Cancer-Associated Fibroblasts Stimulates Epithelial-Mesenchymal Transition and Cancer Stemness [Internet]. Cancer Research. 2010. page 6945–56. Available from: http://dx.doi.org/10.1158/0008-5472.can-10-0785
10. Linxweiler J, Hajili T, Körbel C, Berchem C, Zeuschner P, Müller A, et al. Cancer-associated fibroblasts stimulate primary tumor growth and metastatic spread in an orthotopic prostate cancer xenograft model. Sci Rep. 2020;10:12575.
11. Kalluri R. The biology and function of fibroblasts in cancer. Nat Rev Cancer. 2016;16:582–98.
12. Bostwick DG, Brawer MK. Prostatic intra-epithelial neoplasia and early invasion in prostate cancer. Cancer. 1987;59:788–94.
