# Supplementary material for: Exploring the Onset and Progression of Prostate Cancer through a Multicellular Agent-based Model
Source: Cancer Res Commun. 2023 Aug 7;3(8):1473–85. doi: 10.1158/2767-9764.CRC-23-0097 (PMC10405859; doi:10.1158/2767-9764.CRC-23-0097)
Supplement: Supplementary Table 3 — Parameter values of eight patient groups [file crc-23-0097-s07.pdf]

Supplementary Table 3. *Parameter values of eight patient groups*

**Parameter**	**Low/anti tumor value**	**High/pro tumor value**
TUpmut	0.0000075	0.000175
CFprom	0.01	0.7
M1pmig<br>TUthrshM	0.01<br>20	0.51<br>1

Supplementary Table 3. Model parameter values to distinguish between patients of the eight different categories.
