# Supplementary material for: Exploring the Onset and Progression of Prostate Cancer through a Multicellular Agent-based Model
Source: Cancer Res Commun. 2023 Aug 7;3(8):1473–85. doi: 10.1158/2767-9764.CRC-23-0097 (PMC10405859; doi:10.1158/2767-9764.CRC-23-0097)
Supplement: Supplementary Table 4 — Percentages of cancer in model simulations [file crc-23-0097-s08.pdf]

Supplementary Table 4. *Percentages of cancer in model simulations*

**Simulated patient phenotype**	**Percentage of cancer development**
High CFprom, pro-tumor macrophages & high Pmut	89 %
High Cfprom, anti-tumor macrophages & high Pmut	96%
Low Cfprom, anti-tumor macrophages & high Pmut	96%
Low Cfprom, pro-tumor macrophages & high Pmut	89%
High Cfprom, pro-tumor macrophages & low Pmut	7.5%
Low Cfprom, pro-tumor macrophages & low Pmut	7.9%
High Cfprom, anti-tumor macrophages & low Pmut	27%
Low Cfprom, anti-tumor macrophages & low Pmut	23%

Supplementary Table 4. Percentages of the simulations that resulted in cancer (i.e. break down of the basement membrane) for the eight simulated phenotypes.
