# Supplementary material for: Exploring the Onset and Progression of Prostate Cancer through a Multicellular Agent-based Model
Source: Cancer Res Commun. 2023 Aug 7;3(8):1473–85. doi: 10.1158/2767-9764.CRC-23-0097 (PMC10405859; doi:10.1158/2767-9764.CRC-23-0097)
Supplement: Supplementary Table 5 — Patient markers distinguishing the eight different patient classes [file crc-23-0097-s09.pdf]

***Supplementary Table 5.*** *Patient markers distinguishing the eight different patient classes*

| **Marker** | **Related model parameter** | **Description** | **References** |
|---|---|---|---|
| Macrophage-related markers: below, we describe the patient markers that we chose to determine whether a patient would either have more tumor promoting macrophages (high M2/M1 fraction and high levels of CXCL2 and STAT3) or less tumor promoting macrophages (low patient marker levels). The ratio M1:M2 macrophages indicates whether there are more tumor promoting or anti-tumor macrophages and the CXCL2 and STAT3 expression provide some additional information into the amount of macrophages and their transitioning towards the M2 macrophage. | | | |
| Fraction M2/M1 | *M1pmig TUthrshM* | This fraction describes the ratio of tumor promoting macrophages (M2) to the amount of anti-tumor macrophages (M1). It shows the proportion of macrophages that transitioned towards the tumor-promoting phenotype. | |
| CXCL2 | *M1pmig TUthrshM* | CXCL2 is a chemokine involved in the differentiation process of macrophages towards their pro-tumor phenotype. Indicating whether (many) macrophages are transforming towards the pro-tumor phenotype. | (1,2) |
| STAT3 | *M1pmig TUthrshM* | STAT 3 is a transcription factor involved in the differentiation process of macrophages towards their pro-tumor phenotype. Indicating whether (many) macrophages are transforming towards the pro-tumor phenotype. | (3) |
| Tumor cell related markers: below, we describe the patient markers that relate to the mutation probability. On the basis of these markers, we determined whether a patient had high mutation probability (high mutational burden and high TP53 and CDKN1B) or low mutation probability (low patient marker levels). Mutational burden provides a global quantification of all mutations (including the ones that might not aid PCa progression), while considering also two common tumor-promoting mutations (TP53 and CDKN1B) in PCa provides complementary information on whether the mutations promote PCa progression. | | | |
| Mutational burden | *TUpmut* | An indication of the amount of mutations that have occurred in the tumor cells (this thus is an indication of the frequency with which the patients' cells mutate). | |
| TP53 | *TUpmut* | TP53 is a gene that is commonly mutated in PCa. Especially mutations in exon 7 and 8 contribute greatly to disease progression and recurrence. | (4,5) |
| CDKN1B | *TUpmut* | CDKN1B is a kinase inhibitor that correlates with tumor progression and PSA | (6,7) |

| | | | |
|---|---|---|---|
| | | levels. It is a tumor suppressor gene; loss of heterozygosity has been detected in approximately 50% of prostate tumors. | |
| CAF related markers: below, we describe the patient markers that relate to the tumor promoting ability of CAFs. On the basis of these markers we determined whether a patient had more tumor promoting CAFs (high fraction of CAFs, high TGFBR2 and low IGF1) or less tumor promoting CAFs (low fraction of CAFs and TGFBR2 and high IGF1). We not only studied whether there are many CAFs, but also whether there are pro-differentiation markers (TGFBR2) and their effect of tumor progression (IGF1). | | | |
| Fraction of CAFs | *CFprom* | This fraction describes the number of CAFs present. | |
| IGF1 | *CFprom* | IGF1 is a growth factor secreted by CAFs that plays a role in tumor progression. An inverse relation was found between (the presence of) PCa and levels of IGF1. | (8,9) |
| TGFBR2 | *CFprom* | TGFBR2 is involved in the activation of resident fibroblasts towards CAFs and allows for crosstalk of the tumor microenvironment cells. | (10,11,12) |

*Supplementary Table 5. Patient markers that were used to create the eight different groups of patients. For each class (the first three rows are class: 'TAM', row 4-6 are class 'tumor cells' and row 7-9 are 'CAFs') the three markers were compared to the median value for all included patients. If two (or more) out of three markers were below median value, the patient was marked as 'low' for that class (e.g. low TAM markers means low in terms of TAM, which equals less tumor promoting macrophages). If two (or more) out of three markers were above median value, the patient was marked as 'high' for that class. Combining all the possible combinations (Ranging from all classes high to all classes low) yields the eight different patient phenotypes that were also simulated using the In Silico model. The model parameters that these classes relate to can be found in table S2. M1pmig indicates migration probability of M1 macrophages, TUthrshM indicates the required amount of mutations before a tumor can affect macrophage migration, TUpmut indicates the mutation probability of tumor cells and CFprom indicates the tumor promoting probability by CAFs.*

## *References*


1. Di Mitri D, Mirenda M, Vasilevska J, Calcinotto A, Delaleu N, Revandkar A, et al. Re-education of Tumor-Associated Macrophages by CXCR2 Blockade Drives Senescence and Tumor Inhibition in Advanced Prostate Cancer. Cell Rep. 2019;28:2156–68.e5.
2. Nagarsheth N, Wicha MS, Zou W. Chemokines in the cancer microenvironment and their relevance in cancer immunotherapy. Nat Rev Immunol. 2017;17:559–72.
3. Kumar V, Cheng P, Condamine T, Mony S, Languino LR, McCaffrey JC, et al. CD45 Phosphatase Inhibits STAT3 Transcription Factor Activity in Myeloid Cells and Promotes Tumor-Associated Macrophage Differentiation. Immunity. 2016;44:303–15.
4. Barbieri CE, Baca SC, Lawrence MS, Demichelis F, Blattner M, Theurillat J-P, et al. Exome sequencing identifies recurrent SPOP, FOXA1 and MED12 mutations in prostate cancer. Nat Genet. 2012;44:685–9.
5. Ecke TH, Schlechte HH, Schiemenz K, Sachs MD, Lenk SV, Rudolph BD, et al. TP53 gene mutations in prostate cancer progression. Anticancer Res. 2010;30:1579–86.
6. Lynch SM, McKenna MM, Walsh CP, McKenna DJ. miR-24 regulates CDKN1B/p27 expression in prostate cancer. Prostate. 2016;76:637–48.

7. Xu J, Langefeld CD, Zheng SL, Gillanders EM, Chang B-L, Isaacs SD, et al. Interaction effect of PTEN and CDKN1B chromosomal regions on prostate cancer linkage. Hum Genet. 2004;115:255–62.
8. Cao Y, Lindström S, Schumacher F, Stevens VL, Albanes D, Berndt S, et al. Insulin-like growth factor pathway genetic polymorphisms, circulating IGF1 and IGFBP3, and prostate cancer survival. J Natl Cancer Inst. 2014;106:dju085.
9. Schildkraut JM, Demark-Wahnefried W, Wenham RM, Grubber J, Jeffreys AS, Grambow SC, et al. IGF1 (CA)19 repeat and IGFBP3 -202 A/C genotypes and the risk of prostate cancer in Black and White men. Cancer Epidemiol Biomarkers Prev. 2005;14:403–8.
10. Farhood B, Najafi M, Mortezaee K. Cancer-associated fibroblasts: Secretions, interactions, and therapy. J Cell Biochem. 2019;120:2791–800.
11. De Wever O, Nguyen Q-D, Van Hoorde L, Bracke M, Bruyneel E, Gespach C, et al. Tenascin-C and SF/HGF produced by myofibroblasts in vitro provide convergent pro-invasive signals to human colon cancer cells through RhoA and Rac. FASEB J. 2004;18:1016–8.
12. Lu T, Burdelya LG, Swiatkowski SM, Boiko AD, Howe PH, Stark GR, et al. Secreted transforming growth factor β2 activates NF-κB, blocks apoptosis, and is essential for the survival of some tumor cells [Internet]. Proceedings of the National Academy of Sciences.2004.page7112–7.Availablefrom: http://dx.doi.org/10.1073/pnas.0402048101
